# New Appliances and Things Medical

**Published:** 1893-12-30

**Authors:** 


### NEW APPLIANCES AND THINGS MEDICAL.

[All preparations, appliances, novelties, &c., of which a notice is desired, should be sent for the Editor, to care of The Manager, 428, Strand, London, W.C.]

### ALMOND POUND CAKE.

(Messrs. Callard and Co.)

Messrs. Callard and Co., of Regent Street, have for some time past made a speciality of diabetic foods. These include breads, biscuits, and cakes. A specimen of almond pound cake, which has reached us, will form a pleasant change from the better known compounds. It is light, rich, and very agreeable in taste, and is absolutely free from starch or sugar. Messrs. Callard supply ingredients of all kinds separately, so that those who prefer home-made articles can procure all that is necessary for the purpose.

